# Muscle Co-Contraction Detection in the Time–Frequency Domain

**DOI:** 10.3390/s22134886

**Published:** 2022-06-28

**Authors:** Francesco Di Nardo, Martina Morano, Annachiara Strazza, Sandro Fioretti

**Affiliations:** Department of Information Engineering, Università Politecnica delle Marche, Via Brecce Bianche, 60131 Ancona, Italy; s1107637@studenti.univpm.it (M.M.); a.strazza@pm.univpm.it (A.S.); s.fioretti@staff.univpm.it (S.F.)

**Keywords:** surface EMG signal, co-contraction detection, muscular synergies, the time–frequency domain, wavelet transform

## Abstract

Background: Muscle co-contraction plays a significant role in motion control. Available detection methods typically only provide information in the time domain. The current investigation proposed a novel approach for muscle co-contraction detection in the time–frequency domain, based on continuous wavelet transform (CWT). Methods: In the current study, the CWT-based cross-energy localization of two surface electromyographic (sEMG) signals in the time–frequency domain, i.e., the CWT coscalogram, was adopted for the first time to characterize muscular co-contraction activity. A CWT-based denoising procedure was applied for removing noise from the sEMG signals. Algorithm performances were checked on synthetic and real sEMG signals, stratified for signal-to-noise ratio (SNR), and then validated against an approach based on the acknowledged double-threshold statistical algorithm (DT). Results: The CWT approach provided an accurate prediction of co-contraction timing in simulated and real datasets, minimally affected by SNR variability. The novel contribution consisted of providing the frequency values of each muscle co-contraction detected in the time domain, allowing us to reveal a wide variability in the frequency content between subjects and within stride. Conclusions: The CWT approach represents a relevant improvement over state-of-the-art approaches that provide only a numerical co-contraction index or, at best, dynamic information in the time domain. The robustness of the methodology and the physiological reliability of the experimental results support the suitability of this approach for clinical applications.

## 1. Introduction

Muscle co-contraction is defined as the concurrent activation of agonist and antagonist muscles crossing a targeted joint [1]. It plays a significant role in motion control during physiological activities related to motor learning. Specifically, in able-bodied subjects, co-contraction is a mechanism that occurs to achieve a homogeneous pressure on a joint’s surface, preserving the articular stability and controlling its mechanical impedance [2]. Thus, it occurs frequently in everyday activities such as learning a motor task or handling a tool or an object [3]. In the elderly, injured, and pathological individuals, co-activations play a key role in developing compensation strategies by enhancing joint stability [4,5]. Furthermore, increased co-contraction levels have been detected in orthopedic and neuromuscular patients in order to generate additional joint stiffness to improve joint stability [6,7]. Thus, a quantitative assessment of muscle co-contraction could be considered a meaningful tool that could help us to gain a deeper understanding of how pathology can affect the muscle recruitment strategies during different tasks, including walking.

Several different techniques have been introduced to assess muscle co-contraction, however, a gold standard is not available yet. Advanced mathematical models based on muscle moment assessment have been adopted [8]. Furthermore, simple indexes based on the analysis of electromyography (EMG) signals are potentially very suitable for clinical application [9,10,11,12,13]. Falconer and Winter [13] introduced a co-contraction index (CI) based on the computation of areas under the curve of rectified EMG signals from antagonist muscles. Similar formulations were also reported later [9,10]. Although it is a long-standing index, CI is still commonly held nowadays to provide a rough clinical evaluation of co-contraction [14,15]. However, CI suffers from one main limitation: it provides a single numerical value, which only represents the intensity of the simultaneous muscle activation (the co-contraction level) of each pair of muscles [8]. No information is provided on how long the co-contraction lasts and when it occurs. This information is not clinically negligible, indeed, it has been demonstrated that the modified duration of the co-contraction could be a marker of impairment or could affect the metabolic cost [5,16]. Rudolph et al. tried to overcome this limitation by developing a dynamic co-contraction index that describes the temporal and magnitude components of the EMG signals from antagonist muscles [11]. However, a recent study of young adults showed that Rudolph’s index may present poor reliability during gait [17].

Further studies, including those conducted by the present group of researchers, have quantified muscle co-contraction as the time range where the EMG activity of the two muscles is superimposed [5,18,19]. In this procedure, the onset and offset of a single muscle activity were typically assessed by applying reliable algorithms, such as [20,21]. The overlapping of the activation intervals of the two antagonist muscles provided the time range of the co-contraction. The beginning and the end of this time range have been acknowledged as the onset and the offset of the co-contraction, respectively. All these algorithms reported a good accuracy in assessing the onset and offset of a single muscle’s activity. However, for assessing the co-contraction interval, the algorithm must be applied twice, once for each muscle. Thus, the identification of the co-contraction onset and offset could suffer from the propagation of uncertainty.

To the best of our knowledge, no study is reported in the literature that tries to characterize the frequency content of muscle co-contraction. Typically, frequency analysis is used to quantify the muscle fatigue process in stationary isometric contraction by means of traditional frequency-based techniques, such as Fourier transform [22]. Advanced time–frequency techniques, such as wavelet transform, have been adopted to study the spectral properties of EMG signals over time, trying to characterize the frequency content of muscle activation [23,24]. However, no attempt was made to assess the frequency content of muscle co-contraction.

The purpose of the current work was to propose a novel approach for assessing muscle co-contraction in the time–frequency domain, using the cross-energy localization of the surface electromyographic (sEMG) signals provided by the continuous wavelet transform (CWT) analysis. Specifically, this approach aims to assess a single co-contraction signal and is able to quantify muscular co-contraction in terms of the time interval (onset/offset), the frequency band (maximum–minimum), and the amplitude. CWT is an advanced signal processing technique that maps a time waveform into the time–frequency domain, providing a lossless representation of non-stationary signals in the time and frequency domains. This multiresolution analysis allowed us to define the time–frequency energy density of a signal and to provide a localized statistical assessment of the time–frequency cross-energy density between two signals, i.e., the CWT coscalogram function. The CWT coscalogram was successfully adopted to test the cross-correlation between two different bio-signals [25]. As far as we know, except for a preliminary effort made by this same research group [26], this is the first essay to interpret the CWT coscalogram function between the sEMG signals from two antagonist muscles as the muscular co-contraction activity in the time–frequency domain.

## 2. Materials and Methods

### 2.1. Co-Contraction Detection

A methodology based on continuous wavelet transform (CWT) was adopted to assess muscular co-contraction. CWT is a flexible approach to signal decomposition. CWT is a time–frequency approach, which provides a lossless representation of non-stationary signals in time and frequency domains concomitantly, as represented below:

(1) $$CWT_{sEMG}\left(t,a,b\right)=\int sEMG\left(t\right)\cdot \psi_{a,b}^{*}\left(t\right)\;dt\;\;\;a\neq 0$$

where *sEMG*(*t*) is the input signal and $\psi_{a,b}\left(t\right)$ is the so-called mother wavelet at scale *a* and time *b*, this is represented by Equation (2), as follows:

(2) $$\psi_{a,b}\left(t\right)=\frac{1}{\sqrt{a}}\;\psi \;\left(\frac{t-b}{a}\right)$$

In the present study, CWT analysis was achieved by using the Daubechies of order 4 (factorization in 6 levels) as the mother wavelet. This choice was based on the similarity of Daubechies mother wavelet to the shape of motor unit action potentials [27].

A process for removing noise from the sEMG was performed by applying CWT denoising [28]. The soft Donoho threshold was employed to this aim. The sEMG signal was reconstructed by revised CWT coefficients. Efficacy of the denoising procedure was tested by evaluating the signal-to-noise ratio (SNR) before and after the application of the denoising procedure. SNR was determined by Equation (3), as follows:

(3) $$SNR\left(\sigma_{s},\sigma_{n}\right)=10\times \log\frac{\left(\sigma_{s}^{2}\right)}{\left(\sigma_{n}^{2}\right)}$$

where σ_s_ and σ*_n_* are the signal and noise standard deviation (SD), respectively. The scalogram function $P_{sEMG}\left(a,b\right)$ has been used to represent the energy localization in the time–frequency domain, according to Equation (4), as follows:

(4) $$P_{sEMG}\left(a,b\right)=|W_{sEMG}\left(a,b\right){|}^{2}$$

where *W_sEMG_*(*a*,*b*) is the matrix of CWT coefficients at time *b* and scale *a* for the *sEMG*(*t*) signal. In the present study, the CWT scalogram function in the time–frequency domain of denoised *sEMG* signal was adopted for assessing muscular activation.

**Figure 1:**
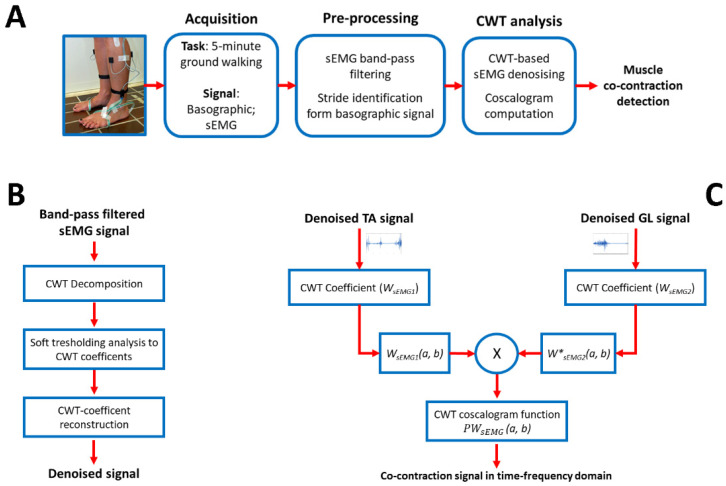
(**A**) Scheme of the entire experimental processing, from data acquisition to muscle co-contraction detection; (**B**) Block diagram representing CWT denoising procedure; (**C**) Block diagram representing the CWT-based procedure for the assessment of muscle co-contraction signals. TA and GL mean tibialis anterior and gastrocnemius lateralis, respectively.

Local cross-correlation between two signals could be identified by computing CWT cross-energy density between signals by means of CWT coscalogram function [25]. For two sEMG signals (*sEMG*_1_(*t*) and *sEMG*_2_(*t*)), CWT coscalogram function, $PW_{sEMG}\left(a,b\right),$ was computed as follows:

(5) $$PW_{sEMG}\left(a,b\right)=W_{sEMG_{1}}\left(a,b\right)\cdot W_{sEMG_{2}}^{*}\left(a,b\right)$$

where $W_{sEMG_{1}}\left(a,b\right)$ and $W_{sEMG_{2}}\left(a,b\right)$ represent the matrices of CWT coefficients of the two denoised signals, at scale *a* and time *b*, the operator * represents the conjugate complex. In the present study, CWT coscalogram function in the time–frequency domain of denoised sEMG signals was adopted for assessing the co-contraction signal between the selected muscles. Co-contraction timing was computed in a single stride as the beginning (onset) and the end (offset) of the time interval when the coscalogram function surpassed 1% of the cross-energy-density peak in the selected stride [29]. Once the co-contraction interval was detected in the time domain, the correspondent co-contraction content in the frequency domain was computed as the frequency range associated with the coscalogram function to that specific time interval. The maximum and minimum of the frequency content were, thus, quantified for each one of the co-contractions assessed in time domain. A block diagram describing CWT procedure for the assessment of the muscle co-contraction signal is presented in Figure 1.

### 2.2. Simulation Study

**Figure 2:**
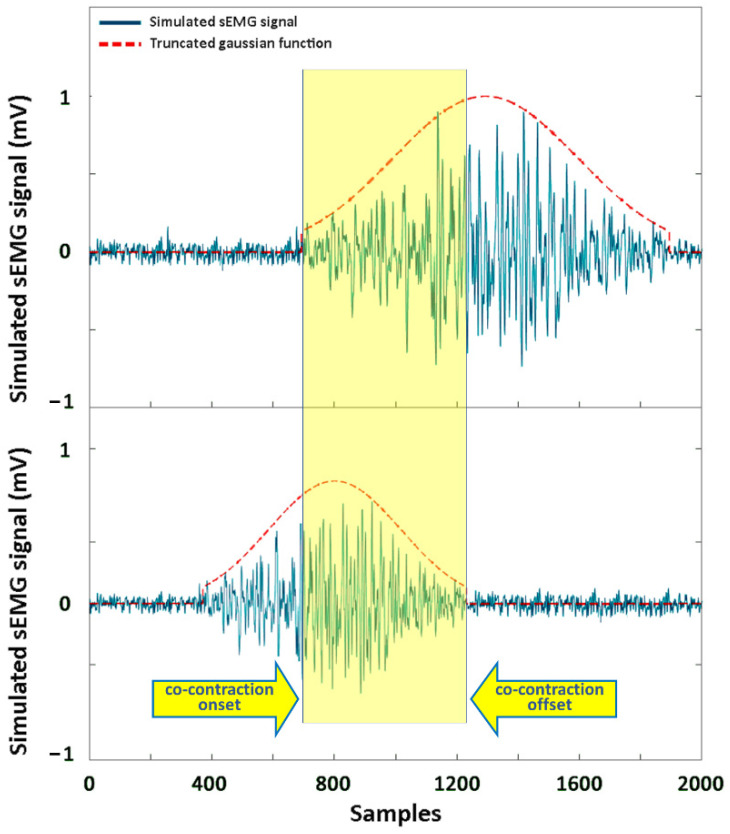
Example of two simulated sEMG signals (in blue), suitable for co-contraction detection. Truncated Gaussian function used to simulate the sEMG activity of each muscle is depicted in dashed red lines. Yellow area represents the co-contraction interval between the two signals. The two yellow arrows indicate the onset and the offset of simulated co-contraction, adopted as ground truth.

A simulation study was conducted for evaluating the performance of the CWT-based procedure in the assessment of the co-contraction signal. It is acknowledged that simulated sEMG signals can be modeled as a process by adding background uncorrelated noise to a bandlimited stochastic process, with zero-mean Gaussian distribution of amplitude and fixed power level, according to [30]. In this study, this distribution was obtained by bandpass filtering a Gaussian series of uncorrelated samples. The bandpass filter cutoff frequencies were 80 and 120 Hz, respectively. This Gaussian distribution was truncated to simulate the sEMG activity due to the muscle activation [30]. Background uncorrelated noise was achieved by a further independent zero-mean Gaussian distribution. Each simulated sEMG signal was generated with a sampling frequency of fs = 1000 Hz, and a time window of =1 s. Different simulated sEMG signals were created by varying the standard deviation, σ, and the time support, 2 × α × σ, of the Gaussian distribution to simulate the physiological variability associated with the recruitment of different muscles. The variation in σ was achieved according to the desired value of the SNR. The following four different SNR values were considered: 5 dB, 10 dB, 15 dB, and 20 dB. Each synthetized signal passed the Anderson’s whiteness test (*p* < 0.05). An example of simulated sEMG signals representing the activity of two muscles is reported in Figure 2.

Performance of the CWT approach for co-contraction detection was evaluated in the time domain by a direct comparison with the ground truth. Specifically, the start and the end of the truncated Gaussian function used to model the simulated signal (Figure 2) were adopted as the ground-truth events for the onset and offset of the activation of a single muscle, respectively. Next, the ground truth for the onset and offset of muscle co-contraction was computed as the beginning and the end of the time interval, where the concomitant presence of the two simulated sEMG signals was detected (yellow area in Figure 2), following the indication reported in [5,18,19]. The beginning sample and the end sample of this co-contraction interval were the ground-truth onset and offset of the co-contraction, respectively. Figure 2 depicts this procedure. Co-contraction interval assessed by the proposed CWT approach was directly compared with the ground-truth co-contraction interval, in terms of onset and offset events, to evaluate the performance of the approach. Results are reported as absolute error (AE) and time delay (TD). AE was computed as the absolute value of the time distance between the predicted event and the corresponding reference event. TD was computed as the relative value (with sign) of the same time distance. Signs “−” and “+” were adopted to indicate that the predicted event occurred earlier and later than the corresponding value in the reference signal, respectively.

### 2.3. Experimental Study

Experimental data included the foot–floor contact and sEMG signals collected during the walking of 30 healthy young adults, which was retrospectively taken from the dataset built up at Movement Analysis Lab, Università Politecnica delle Marche, Ancona, Italy. The data are freely available by consulting the public repository of medical research data PhysioNet [31,32,33]. Subjects with a body mass index (BMI) lower than 18 kg/m^2^ (underweight) and higher than 25 kg/m^2^ (overweight) were ruled out from the investigation. Patients who communicated manifest disorders, diseases, pain, or who had undergone surgical intervention were kept out of the considered population. 

The acquisition system Step32 (Medical Technology, Turin, Italy) was used to acquire all the walking signals. A sampling rate of 2000 Hz and a resolution of 12 bit were adopted for the acquisition procedure. Differential sensors were employed to measure sEMG signals over the following two ankle muscles of each leg during 5 min of ground walking: the gastrocnemius lateralis (GL) and the tibialis anterior (TA). The procedure of positioning the sEMG probes was conducted according to the SENIAM recommendation for sensor locations on muscles in the lower leg and foot [34]. Characteristics of the probes were as follows: single differential probes with fixed geometry, constituted by Ag/Ag-Cl disks; size—7 mm × 27 mm × 19 mm; electrode diameter—4 mm; interelectrode distance—12 mm; gain—1000; high-pass filter—10 Hz; and input impedance, >1.5 GΩ, CMRR > 126 dB. The sEMG signals were bandpass filtered (20–450 Hz) and then processed as reported in Section 2.1. CWT scalogram and coscalogram were computed separately in every single stride.

Footswitches, applied on the sole of each foot (heel, first, and fifth metatarsal heads), were utilized to synchronously capture basographic data during the same task. An eight-level coded basographic signal was acquired from three foot switches (size: 11 mm × 11 mm × 0.5 mm; activation force: 3 N), which were beneath the heel, first, and fifth metatarsal heads of each foot. Foot-switch signals were converted to the following four levels: heel contact (H), flat foot contact (F), push off (P), and swing (S). They were processed to identify the beginning and the end of each stride, i.e., the gait cycle [35]. Subjects walked barefoot on level ground for approximately 5 min at their natural speed and pace, following an eight-shaped path, which included rectilinear segments and curves. This study was fulfilled observing the ethical principles of the Helsinki Declaration and was approved by the local ethical committee.

Unlike the simulated signals, no ground truth was available for the experimental dataset. Thus, the direct computation of detection accuracy, AE, and TD was not possible. Thus, the performances of the proposed CWT approach have been evaluated in the time domain by a direct comparison with the outcomes achieved by using a reference approach. Reference values were assessed as follows: The onset and offset timing of TA and GL activity was identified on denoised sEMG signals by applying the double-threshold statistical detector (DT), an approach particularly suitable for application in the walking task [36]. Onset and offset of co-contraction intervals were computed as the beginning and the end of the superimposition between the activation intervals, assessed by DT, for TA and GL in the same stride. These values were adopted as reference values of the co-contraction onset and offset.

### 2.4. Statistics

The significant difference of the parameter distribution was determined through the following statistical tests. The normality of each distribution was tested by adopting the Shapiro–Wilk test. The possible significance of the statistical difference was tested for the following: (1) for normal distributions by means of the two-tailed, non-paired Student’s *t*-test, between two distributions and using analysis of variance (ANOVA) for multi-group comparison, and (2) for non-normal distributions through the Mann–Whitney test, between two distributions and through Kruskal–Wallis’ test for multi-group comparison. 5% was the threshold adopted for detecting the test significance.

## 3. Results

### 3.1. Simulation Study

**Figure 3:**
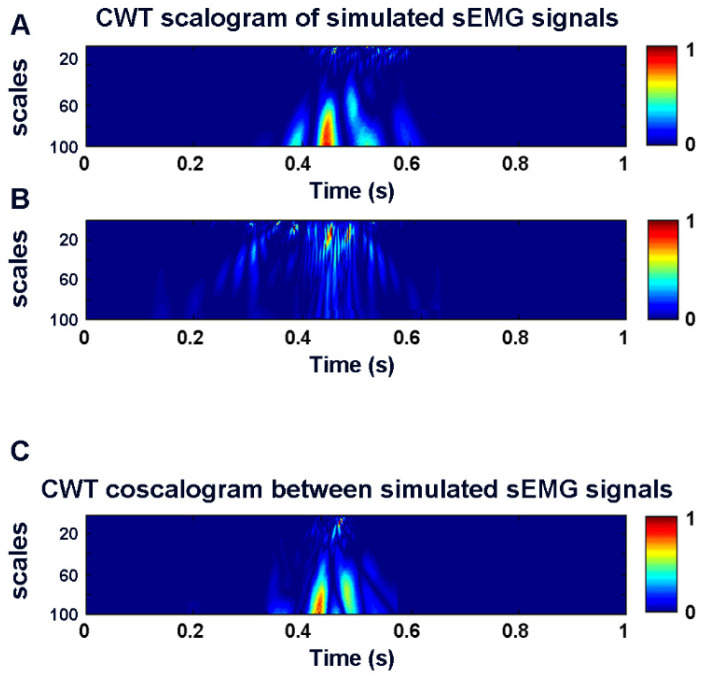
Two-dimensional color representation of CWT scalogram function for two simulated sEMG signals (**A**,**B**). (**C**) shows the CWT coscalogram between the simulated sEMG signals in (**A**,**B**).

Figure 3 shows the two-dimensional color representation of the CWT scalogram function for the two simulated sEMG signals (panel A and B, respectively). Panel C of the same figure depicts the cross-energy density in the time–frequency domain between the denoised simulated sEMG signals, represented by the CWT coscalogram function, i.e., the estimated co-contraction signal.

**Figure 4:**
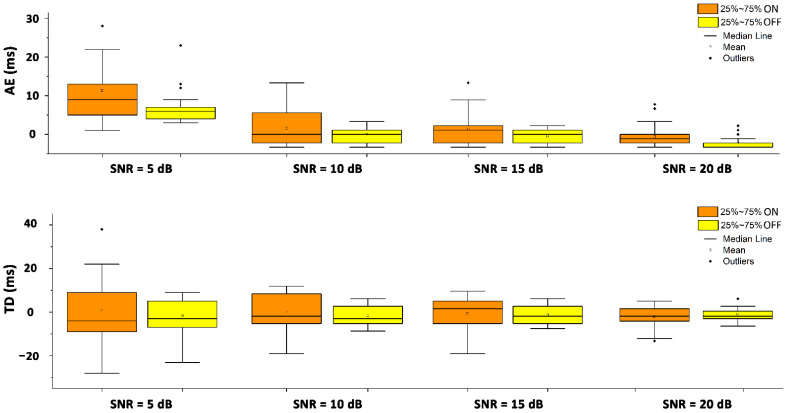
Whisker plots of absolute error (AE, upper panel) and time delay (TD, lower panel) computed over simulated sEMG co-contractions per SNR value expressed in dB. Orange bars represent the onset values. Yellow bars represent the offset values. The AE values computed for SNR = 5 dB were significantly higher (*p* < 0.05) than all the AE values computed in the other three SNR conditions for onset and offset instants of the co-contraction.

The accuracy in predicting the co-contraction onsets and offsets in the whole dataset of the simulated sEMG signals was 100%. This means that the proposed CWT approach was able to identify all the simulated co-contraction intervals (recall = 100%) and that no false positives were detected (precision = 100%). The prediction errors for the co-contraction onset and offset were quantified in terms of AE and TD. The mean, SD, median, 25th percentile, and 75th percentile for each SNR value are depicted in Figure 4 and further reported for the TD in Table 1. The AE values decreased with an increase in the SNR from 5 to 20 dB. The mean and median TD values for both the onset and offset fell in the [−5 ms; 5 ms] range for all the SNR values. The AE values computed for SNR = 5 dB were significantly higher (*p* < 0.05) than all the AE values computed in the other three SNR conditions for the detection of both the onset and offset instants of the co-contraction.

### 3.2. Experimental Study

Detailed values of the SNR for raw sEMG signals (SNR raw) and for sEMG signals after CWT denoising (SNR denoised) for the whole population are reported in Table 2. The mean, SD, median, 25th percentile, and 75th percentile are reported at the bottom of the table.

**Figure 5:**
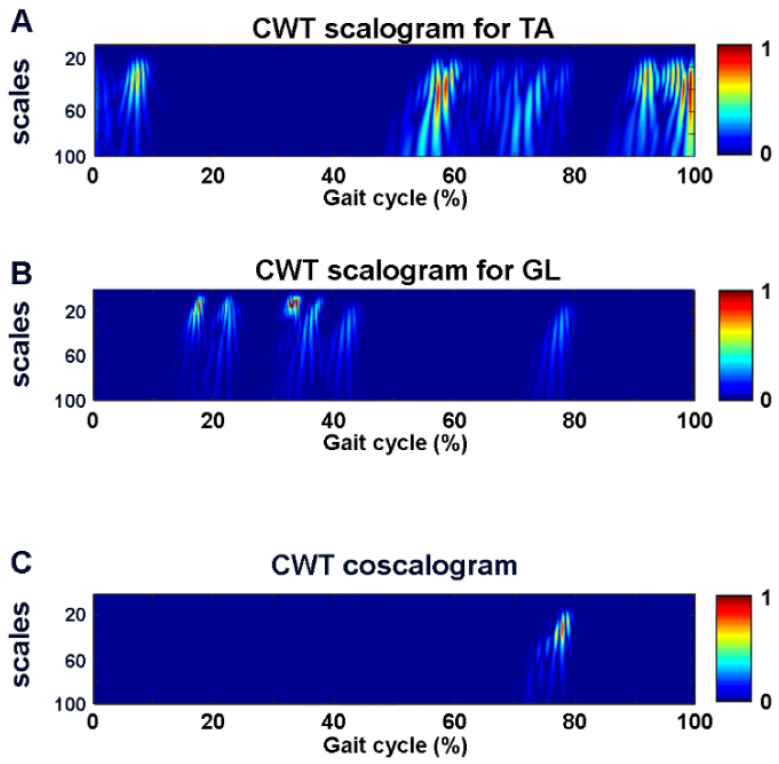
Two-dimensional color representation of CWT scalogram function for TA (**A**) and GL (**B**) denoised sEMG signals and CWT coscalogram between TA and GL (**C**) for a representative stride from subject five.

**Figure 6:**
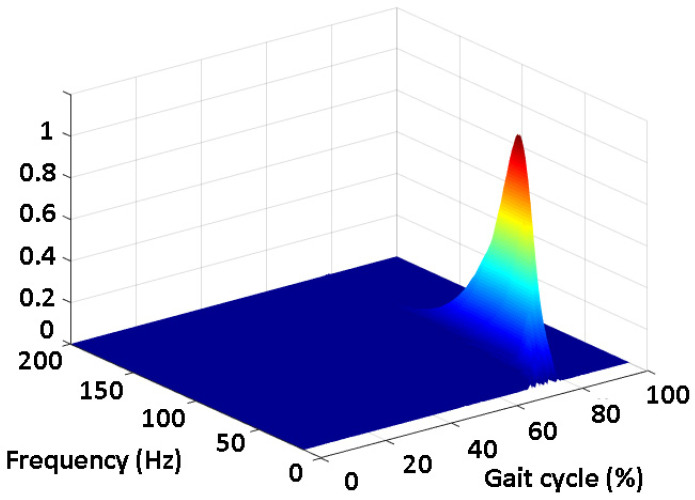
Three-dimensional color representation of CWT coscalogram between TA and GL for the same representative stride of subject five, depicted in Figure 5.

An increase in the SNR value after the denoising procedure was reported in each single signal. An SNR improvement (*p* < 0.05) was detected for both the TA and the GL in terms of the mean, SD, median, 25th percentile, and 75th percentile value. The two-dimensional color representation of the CWT scalogram function for the TA (panel A) and the GL (panel B) denoised sEMG signals is reported in Figure 5, for a representative stride from subject five (low SNR). Panel C of the same figure shows the cross-energy density in the time–frequency domain between the denoised TA and GL signals, represented by the CWT coscalogram function, i.e., the estimated co-contraction signal in a representative stride in the walking task. The 3D representation of the CWT coscalogram function for the same stride is depicted in Figure 6.

**Figure 7:**
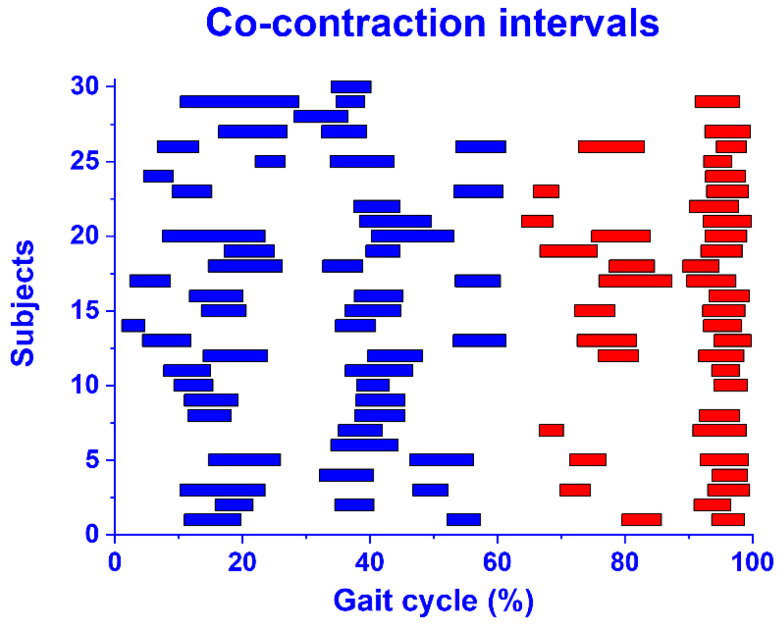
All the average co-contraction intervals detected in stance (blue bars) and in swing (red bars) in each one of the thirty subjects of the population. Values are expressed in percentage of gait cycle.

The average co-contraction intervals achieved in each subject by the application of the proposed CWT approach are illustrated as horizontal bars in the percentage of gait cycle, in Figure 7. The co-contractions detected in the stance and swing phase are highlighted in blue and red, respectively.

**Figure 8:**
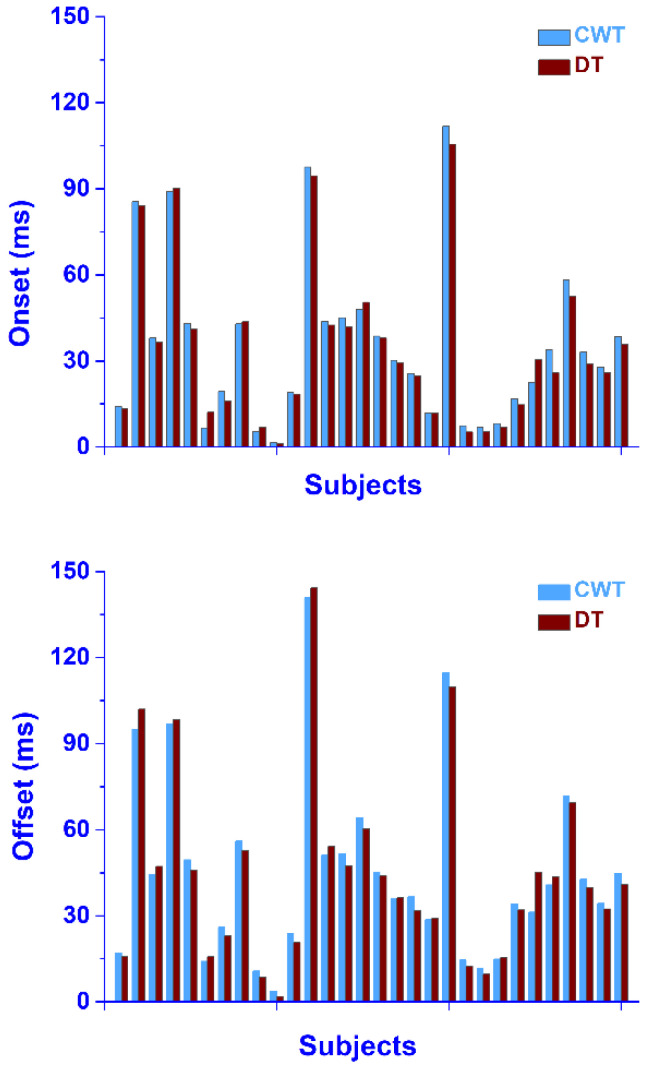
Average co-contraction onset and offset detected in early stance by CWT (cyan bars) vs. DT (brown bars) on real sEMG signals collected during walking. Values are expressed in ms, as the time–distance from the previous heel strike.

The performance of the CWT approach in providing the co-contraction onset and offset was assessed by a direct comparison with the DT algorithm in the experimental sEMG signals from thirty subjects, including a total of 16,315 strides, resulting in at least 100 co-contraction bursts per subject. The results for each subject are reported in Figure 8. No significant differences were identified between the average values over the whole population, achieved by the two approaches for the co-contraction onset and offset. Furthermore, no significant difference was detected in each single subject (*p* > 0.05, Figure 8).

**Figure 9:**
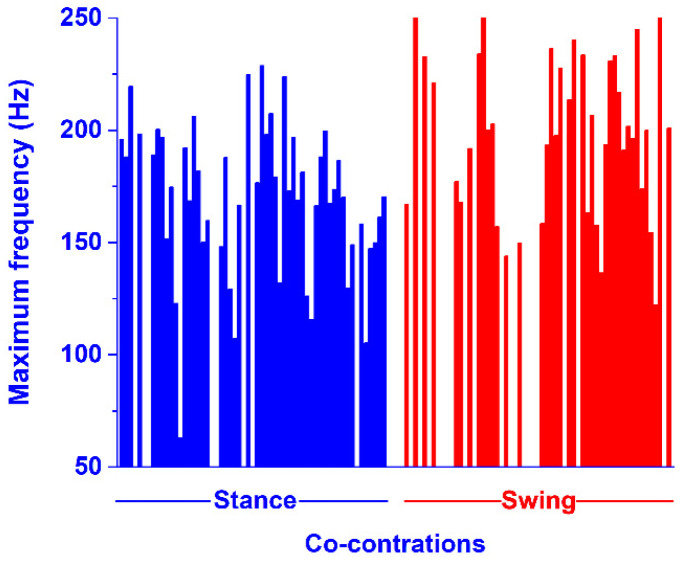
Average values of the maximum frequency computed in every co-contraction detected in time domain, as reported in Figure 7. Blue and red bars represent the maximum frequency of the co-contraction detected in stance and swing, respectively. Each single bar represents the average value of the maximum frequency detected for each co-contraction in a single subject.

**Figure 10:**
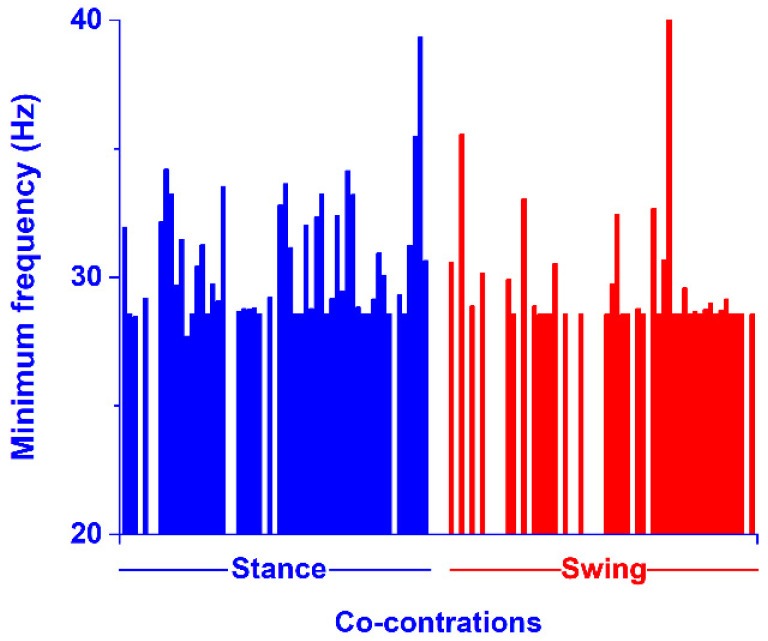
Average values of the minimum frequency computed in every co-contraction detected in time domain, as reported in Figure 7. Blue and red bars represent the minimum frequency of the co-contraction detected in stance and in swing, respectively. Each single bar represents the average value of the minimum frequency detected for each co-contraction in a single subject.

The current CWT algorithm was also able to provide the frequency content of each one of the co-contractions detected in the time domain, as the frequency of the coscalogram signal in the specific time range where the co-contraction was detected. The values of the maximum frequency of each co-contraction signal are shown in Figure 9. Specifically, each bar in Figure 9 represents the maximum frequency of each one of the co-contractions represented in Figure 7. This was computed as the average value over all the maximum frequency values assessed in each stride, where the co-contraction was detected in that specific subject. For example, the first blue bar in Figure 9 is the average maximum frequency computed in the first co-contraction of subject one (in Figure 7); the second blue bar is the average maximum frequency computed in the first co-contraction of subject one (in Figure 7), and so on. The white space between the colored bars represents the absence of a specific co-contraction in a subject (in stance or in swing). The values of the minimum frequency of each co-contraction signal, computed as for the maximum frequency, are shown in Figure 10. The separation between the co-contractions in stance and swing is still respected here, as in Figure 7.

## 4. Discussion

The current investigation was designed to propose a novel approach for muscle co-contraction detection, based on the CWT cross-energy localization of two sEMG signals in the time–frequency domain, i.e., the CWT coscalogram. As far as we know, this is the first thorough study that has tried to adopt the CWT coscalogram between two sEMG signals from antagonist muscles in order to characterize the muscular co-contraction activity in the time–frequency domain. The CWT coscalogram allowed us to compute a single co-contraction signal, by which it was possible to concomitantly assess the timing of the co-contraction occurrence, its frequency content, and its amplitude.

In the field of sEMG data analysis, sEMG signals have been processed in the time–frequency domain, based on wavelet transform technology. This has also been conducted with the aim of assessing muscle co-contractions and synergies. Lee et al. [37] achieved an sEMG-based estimation of lower limb muscle co-contractions in patients with incomplete spinal cord injury. A wavelet analysis was used to process the signal in order to compute the sEMG signal intensity. Furthermore, the principal component analysis was adopted to characterize the co-contraction. The outcomes were reported in terms of correlation, however, the time-duration and frequency content were not computed and the detection accuracy was not reported. Du et al. [38] attempted to discriminate the possible difference of lumbar muscles’ co-contraction in patients affected by lumbar disc herniation. A wavelet analysis was performed to filter the sEMG signals, while classic index-based time–domain methods were used to assess the co-contraction between filtered sEMG signals. The results were provided in terms of a simple index and a co-contraction ratio, however, the time-duration and frequency content were not assessed and the detection accuracy was not reported. Frere et al. tried to assess the synergy of upper-limb muscles in gymnasts [39]. A wavelet analysis was only used to process the signal in order to extract the sEMG envelopes suitable to compute the muscle synergies. The actual computation of the synergies was achieved by adopting the traditional non-negative matrix decomposition method. Similarly, Xie et al. [40] identified muscle synergy in wrist motion by means of a time–frequency approach. A wavelet packet transform was used to decompose and characterize the sEMG signals in each specific frequency band. Synergy modules were extracted by applying the non-negative matrix factorization method in each frequency band. In all these approaches, the wavelet technique was only adopted with the aim of processing and filtering the sEMG signals. Other techniques (e.g., non-negative matrix factorization, NMF, principal component analysis, PCA, and classic index-based approaches) have been used to provide quantitative information on co-contraction. Moreover, NMF methods are only appropriate in assessing the synergies between a certain number of muscles, as they do not work for the identification of the synergy between two muscles, i.e., the co-contraction. Thus, to the best of our knowledge, none of the wavelet-based attempts to characterize muscle co-contraction have been able to provide a quantitative concomitant assessment of co-contraction time duration and frequency content. Conversely, this present study provides the timing, magnitude, and frequency content of muscle co-contraction concomitantly. In addition, the current approach not only allowed us to achieve the frequency content of the whole co-contraction signal, but also of the frequency band of each single occurrence of co-contraction in the gait cycle. These considerations summarize the main contributions of the study.

Although no attempt has been made to identify the frequency content, some approaches in the time domain are available to assess co-contraction timing [5,9,10,11,12,13,18,19]. However, the only studies able to provide co-contraction timing (i.e., the onset and offset of muscle co-contraction) are those based on the identification of muscle co-contraction as the time range where the sEMG activity of the two muscles is superimposed [5,18,19]. The overlapping of the activation intervals of the two antagonist muscles provides the time range of co-contraction. The beginning and the end of this time range is acknowledged as the onset and the offset of the co-contraction, respectively. Thus, the proposed CWT approach has been validated against those studied by a direct comparison of the real and simulated co-contraction onset and offset identified in the time domain.

The performances of the CWT approach in the detection of muscular co-contraction were directly estimated in both the synthetic and real sEMG signals. For the simulation study, since no reference data or technique were available in the time–frequency domain, validation was performed in the time domain, where co-contraction is typically quantified as the temporal interval where the sEMG activity of the two muscles is superimposed [5,18,19]. Essentially, the direct comparison of the co-contraction interval provided by the CWT approach was performed with the time interval, where the two clean, simulated sEMG signals were superimposed (ground truth). The accuracy in predicting the co-contraction onsets and offsets in the whole dataset of the simulated sEMG signal was 100%. Furthermore, the mean error with respect to the ground truth (as defined in Section 2.3) was computed in terms of AE and TD (±SD) (Figure 4). These results of the assessment of the co-contraction between two muscles are comparable with those achieved for the detection of the activation of a single muscle in the aforementioned recent studies [20,21,29,36,41]. However, it is worth highlighting that each one of the aforementioned studies reported the performance of the algorithm in the assessment of the activity of a single muscle. For assessing co-contraction onset and offset, the algorithm should be applied twice, once for each muscle. Thus, a problem could arise with the propagation of the error and a larger bias could occur. This issue does not concern the present CWT approach because it works on a single co-contraction signal. Moreover, the accuracy of the CWT approach could be considered widely satisfactory within the tested SNR range of the simulated sEMG signals. A statistical analysis showed that, for SNR values >5 dB, the performance of the CWT approach in the detection of both onset and offset instants did not significantly change (Figure 4). This result matched with [20]. The significant increase in the AE detected for SNR = 5 dB (*p* < 0.05, upper panel in Figure 4) indicated a worsening of the detector performance for low SNR values. This worsening, however, did not affect the reliability of the detection, since the AE values are comparable with values reported by others [20,41] for similar or higher SNRs and for the activity of a single muscle. The TD values lower than 5 ms in the whole SNR range support the trustworthiness of the prediction and show that the estimates are not polarized (lower panel in Figure 4).

The CWT approach was also tested on a dataset of the experimental sEMG signals from ankle antagonist muscles (walking data from 30 able-bodied subjects). As indicated in [42], a wavelet approach guarantees a reliable denoising of the sEMG signals. In the present population, the denoising procedure allowed a significant improvement (*p* < 0.05) in the average SNR of 34.5% for TA and 33.6% for GL (Table 2), leading to an average increase in the SNR from 14.5 ± 7.0 dB to 19.5 ± 8.0 dB for TA, and from 14.0 ± 5.5 dB to 18.7 ± 7.1 dB for GL. This provided more reliable ankle muscle sEMG signals (reduced noise) for the subsequent co-contraction quantification. Figure 5 shows that the application of the CWT approach to real signals was able to represent the co-contraction between the TA and GL activity in the functions of time (*x*-axis), frequency (*y*-axis), and magnitude (colored scale). In particular, this representation allowed a simple and direct identification of co-contraction in the time domain (% of gait cycle). A 3D graph (Figure 6) appeared to be more suitable for an overall description of the co-contractions, including the magnitude and frequency bands. A graphical representation of all the co-contractions detected in the 30-subject population used in this study is reported in Figure 7. This graph allows us to identify the main zones where co-contraction occurs and the occurrence rate of this co-contraction. The outcomes are consistent with the results reported by the referenced studies on ankle muscle co-contractions, detected in numerous strides (more than ten thousand), with a more traditional approach (i.e., the superimposition of activation intervals between two muscles) [19]. Indeed, the occurrence of co-contraction in early stance, during weight acceptance, and in the final swing were detected both in [19] and in the current analysis, supporting the suitability of the current detector for experimental applications. The high occurrence rate during the late swing (Figure 7) is also in agreement with the results reported in [19]. A further validation of the results provided by the CWT approach in the time domain was performed by means of a direct comparison with a reference co-contraction interval and assessed as the temporal interval where the sEMG activity of the two muscles were superimposed, as described in Section 2.3. No gold standard is available for the detection of muscular onset and offset during walking. In the present study, Bonato’s double-threshold (DT) statistical detector was used as a reference [36], due to its acknowledged reliability in assessing muscular onset and offset [43]. Since no ground truth was available for this dataset, the computation of the detection accuracy was not possible. However, the reliability of the present approach is supported by the fact that the two approaches (CWT and DT) identified the same number of co-contraction intervals in the whole experimental sEMG dataset. The outcomes of the co-contraction onset and offset that were assessed in real sEMG signals with SNR (after denoising), ranged from 4.3 dB to 39.1 dB (as shown in Table 2) and are reported in Figure 8. The performance of each of the two detectors in providing the co-contraction onset and offset were comparable, since the small difference highlighted in Figure 8 was not statistically significant (*p* > 0.05). This suggests the trustworthiness of the present results in the time domain.

However, the actual novel contribution of the current CWT approach consists of providing the frequency values of each muscle co-contraction detected in the time domain, as the frequency of the coscalogram signal in the specific time range where the co-contraction is detected. To the best of our knowledge, this is the first attempt that have been made to achieve this. This represents a relevant improvement over the state-of-the-art approaches that provide only a numerical co-contraction index [8,9,10,13,14] or, at best, dynamic information in the time domain [4,11,17,18,19], since it has been shown that frequency content could be used to reveal changes in the electrophysiological characteristics associated to specific disorders of the neuromotor system [44]. Figure 9 shows the distribution of the maximum values of the frequency content provided by the CWT algorithm in the present population, separately for the stance and swing phases. The figure highlights a wide variability in the frequency maximum between the subjects and within the strides. In particular, co-contraction seems to assume higher average and peak values of frequency in swing, compared to stance. Minimum frequency variability was also detected, mainly during the stance phase (Figure 10). A plausible interpretation of this phenomenon could be related to the functional/control tasks of the different co-contraction occurrences during the gait cycle. Even though the quantitative analysis of co-contraction frequency content is beyond the purpose of the current research project, these outcomes raise a novel question that deserves to be investigated. New and specific tools, such as the approach introduced by the current study, could be beneficial to this goal.

In conclusion, the current study proposes the CWT coscalogram function between the sEMG signals from antagonist muscles as a suitable tool to assess the muscular co-contraction activity in the time–frequency domain. This CWT approach successfully provided an overall characterization of the co-contraction phenomenon with a 3D range of variability (time, magnitude, and frequency), allowing us to monitor possible changes of this range to correlate the relative role of each one of the 3D components (time, magnitude, and frequency) in the phenomenon. This approach could be particularly valuable in clinical and rehabilitation environments, since changes in the co-contraction picture of a subject was acknowledged as a marker of neurological impairment [5], meaning that patients could be adequately encouraged to obtain positive long-term clinical outcomes [45]. The robustness of the methodology, the satisfactory accuracy, the precision in co-contraction detection, and the physiological reliability of the experimental results further support the suitability of the present tool for clinical applications. In the current paper, the performance of the CWT approach was validated on simulated signals and on real sEMG signals, which were acquired during walking. Future studies should focus on a direct validation of different motor tasks, such as jumping, squatting, and running.

## Figures and Tables

**Table 1:** Time delay (TD) ± SD computed over simulated sEMG co-contractions per SNR value, expressed in dB.

	SNR (dB)	Mean (ms)	SD (ms)	Median (ms)	25th Percentile (ms)	75th Percentile (ms)
Onset	5	0.52	14.6	−4.0	−9.1	9.2
	10	0.96	6.9	−1.2	−4.0	8.1
	15	0.10	6.4	2.1	−4.3	5.2
	20	−1.16	4.3	−1.4	−3.2	2.4
Offset	5	−1.80	7.9	−3.1	−7.0	5.4
	10	0.76	4.3	−2.2	−4.2	3.3
	15	0.52	4.0	−1.2	−4.1	3.0
	20	0.20	2.5	−1.0	−2.1	1.4

**Table 2:** SNR of the raw sEMG signals (SNR raw) and after CWT denoising (SNR denoised) for tibialis anterior and gastrocnemius lateralis.

	Tibialis Anterior		Gastrocnemius Lateralis	
Subject	SNR Raw	SNR Denoised	SNR Raw	SNR Denoised
1	8.9	17.7	13.1	39.1
2	4.7	8.5	5.8	17.6
3	13.3	17.5	16.4	17.5
4	9.8	12.7	5.0	7.7
5	7.3	9.1	8.7	10.5
6	12.6	19.2	16.1	18.5
7	24.7	30.8	10.7	15.4
8	23.5	32.3	20.5	33.0
9	4.5	9.3	3.3	7.3
10	2.4	4.3	6.0	8.2
11	27.3	35.4	16.3	21.7
12	22.1	29.7	11.3	16.8
13	17.2	21.7	13.1	19.4
14	16.4	19.3	18.5	20.7
15	5.5	9.8	13.9	18.5
16	18.1	24.3	14.9	15.4
17	25.3	32.9	17.4	20.5
18	27.1	31.2	25.8	30.7
19	11.5	18.1	12.7	15.1
20	16.2	18.9	17.0	20.1
21	14.2	16.7	18.1	19.4
22	12.2	16.4	13.9	15.8
23	20.2	25.6	12.9	17.4
24	11.8	20.9	18.9	21.7
25	13.1	18.8	21.0	24.4
26	17.8	19.9	20.5	23.7
27	14.6	17.6	15.6	18.3
28	5.4	9.8	3.9	8.5
29	17.3	20.5	12.5	15.1
30	10.6	15.7	16.8	23.1
Mean	14.5	19.5	14.0	18.7
SD	7.0	8.0	5.5	7.1
Median	13.8	18.9	14.4	18.4
25th percentile	9.8	15.7	11.3	15.4
75th percentile	18.1	24.3	17.4	21.7

All the values are expressed in dB. SNR—signal-to-noise ratio. SNR distributions after denoising (SNR denoised) were significantly different (*p* < 0.05) from the corresponding value of the SNR raw, for both TA and GL.

## Data Availability

Data are freely available by consulting the public repository of medical research data, PhysioNet, at the following link: https://physionet.org/content/semg/1.0.0/ (accessed on 30 May 2022).
